# New Books

**Published:** 2004-11

**A Scientist Audits the Earth**

Stuart L. Pimm

Piscataway, NJ:Rutgers University Press, 2004. 304 pp. ISBN: 0-8135-3540-9, $19.95

**Academia to Biotechnology: Career Changes at any Stage**

Jeffrey Gimble

Burlington, MA:Elsevier, 2004. 186 pp. ISBN: 0-12-284151-4, $34.95

**Acid Rain Science and Politics in Japan: A History of Knowledge and Action toward Sustainability**

Ken Wilkening

Cambridge, MA:MIT Press, 2004. 328 pp. ISBN: 0-262-73166-5, $19

**Cells, Tissues, and Disease: Principles of General Pathology, 2nd ed.**

Guido Majno, Isabelle Joris

New York:Oxford University Press, 2004. 1040 pp. ISBN: 0-19-514090-7, $198.50

**Correction Lines: Essays on Land, Leopold, and Conservation**

Curt Meine

Washington, DC:Island Press, 2004. 304 pp. ISBN: 1-55963-732-3, $25

**Encyclopedia of Ecological and Environmental Toxicology**

Jason Weeks, Michael C. Newman, Sheila O’Hare

Hoboken, NJ:John Wiley & Sons, Inc., 2004. 2000 pp. ISBN: 0-471-49559-X, $1,200

**Environmental Governance Reconsidered: Challenges, Choices, and Opportunities**

Robert F. Durant, Daniel J. Fiorino, Rosemary O’Leary, eds.

Cambridge, MA:MIT Press, 2004. 536 pp. ISBN: 0-262-54174-2, $35

**Global Institutions and Social Knowledge**

Virginia M. Walsh

Cambridge, MA:MIT Press, 2004. 208 pp. ISBN: 0-262-73167-3, $20

**Imitation of Life How Biology Is Inspiring Computing**

Nancy Forbes

Cambridge, MA:MIT Press, 2004. 176 pp. ISBN: 0-262-06241-0, $25.95

**Introduction to Environmental Toxicology: Impacts of Chemicals Upon Ecological Systems, 3rd ed.**

Wayne G. Landis, Ming-Ho Yu

Boca Raton, FL:CRC Press, 2004. 484 pp. ISBN: 1-56670-660-2, $99.95

**Live Cell Imaging: A Laboratory Manual**

Robert D. Goldman, David L. Spector, eds.

Woodbury, NY:Cold Spring Harbor Laboratory Press, 2004. 648 pp. ISBN: 0-87969-682-6, $250

**Managing Scientists: Leadership Strategies in Scientific Research, 2nd ed.**

Alice M. Sapienza

Hoboken, NJ:John Wiley & Sons, Inc., 2004. 246 pp. ISBN: 0-471-22614-9, $39.95

**Recombinant Gene Expression: Reviews and Protocols, 2nd ed.**

Paulina Balbas, Argelia Lorence

Totowa, NJ:Humana Press, 2004. 494 pp. ISBN: 1-58829-262-2, $125

**Regulators of G Protein Signalling, Part B (Methods in Enzymology, Vol. 390)**

David Siderovski

Burlington, MA:Elsevier, 2004. 560 pp. ISBN: 0-12-182795-X, $149.95

**Research Proposals: A Guide to Success, 3rd ed.**

Thomas Ogden, Israel Goldberg

Burlington, MA:Elsevier, 2004. 368 pp. ISBN: 0-12-524733-8, $34.95

**Scientific Papers and Presentations, 2nd ed.**

Martha Davis

Burlington, MA:Elsevier, 2004. 384 pp. ISBN: 0-12-088424-0, $29.95

**Sertoli Cell Biology, Vol. 1**

Michael K. Skinner, Michael D. Griswold, eds.

Burlington, MA:Elsevier, 2004. 512 pp. ISBN: 0-12-647751-5, $229.95

**The Proteus Effect: Stem Cells and Their Promise**

Ann B. Parson

Washington, DC:National Academies Press, 2004. 256 pp. ISBN: 0-309-08988-3, $22.45

**Welcome to the Genome: A User’s Guide to the Genetic Past, Present, and Future**

Rob DeSalle, Michael Yudell, American Museum of Natural History

Hoboken, NJ:John Wiley & Sons, Inc., 2004. 215 pp. ISBN: 0-471-45331-5, $29.95

